# Supplementary material for: Community responses to a novel house design: A qualitative study of “Star Homes” in Mtwara, southeastern Tanzania
Source: PLoS One. 2025 Jan 22;20(1):e0309518. doi: 10.1371/journal.pone.0309518 (PMC11753654; doi:10.1371/journal.pone.0309518)
Supplement: S2 Table — (PDF) [file pone.0309518.s002.pdf]

STUDY TITLE: STAR HOMES PROJECT **COMPONENT**: SOCIAL SCIENCE
DATA COLLECTION GUIDE TYPE: H-HH IDI GUIDE v2

A: STUDY-SPECIFIC DETAILS

VILLAGE NAME: |____________________| INTERVIEW DATE: |_|_|/|_|_|/|_|_|_|_|

STUDY HOUSE IDNo: |__|__|__| RESPONDENT CATEGORY: H-HH / NON-H-HH

SS INTERVIEWER INITIALS: |__|__|__| **START TIME: |___|___|:|___|___| am/pm**

B: INFORMED CONSENT PROCEDURES

Remind the respondent about the use of a tape recorder and turn it ON now!

C: SOCIO-DEMOGRAPHIC DETAILS

RESPONDENT INITIALS	AGE GROUP	SEX	EDUCATION (YEARS)	ROLE/LIVELIHOOD ACTIVITY

D: THE INTERVIEW

TOPIC	SUGGESTED QUESTIONS AND PROBES
**CONSENT CONFIRMATION**	Have you consented to participate in this interview? Yes/No (*If no, end here*)
**WARM UP (brief if interviewed before)**	• What do you understand by Star homes/houses? (**In Swahili**: *mradi wa CSK/Nyumba za CSK*)?<br>• As a head of HH, how did you get a star house? Were you satisfied by the process that led you to get a star house? If not, why?<br>• When (date, month and year) did you get the house?<br>• Are you living in the house now?<br>• When did you start to live in the house? (If not immediately after getting a house) Why?<br>• In total, how many children (<13) are there in your household?<br>• Did you hear of any community engagement activities about the homes (e.g. radio show, football patches)? Did you participate? What did you think about them?

**OPINIONS ON OVERALL HOUSE DESIGNS (brief if interviewed before)**	• Please tell me, what is your overall opinion of the house?<br>• What do you like about the house?<br>• What do you dislike about the house? **Probe:** Rooms? Toilet/latrines? What else?<br>• If you could change anything? What would that be? Why would you do that?<br>• Before moving into the house, what concerns did you have about the move?" [***Interviewer:*** *Take note of, secret rooms, blood sacrifice, freemason, death of children and the like myths*] Where did you get the concern(s) from?<br>• Now that you are living in the house, what concerns do you have about the house [if different from the time before moving into the house]? The rooms? Toilet/latrines?<br>• Since you started living in the home, what do you do different? E.g. Good to sleep at a different time? Eat more inside? What is it that makes you do such things differently?<br>• Do you sleep in the house occasionally? If yes, why? **OR.**<br>• Do you sleep in the house every night? If not, why not?<br>• Do all members of your family sleep in the house? If not, why not? (if not mentioned, probe for children)<br>• How many children (<13) are currently living in the house?<br>• How many children (<13) are currently **NOT** sleeping in the house?
**PERCEIVED COMMUNITY IMPRESSION (brief if interviewed before)**	• What do your neighbors (who are not living in the project houses) say about the house? [***Interviewer:*** *Encourage the respondent to mention all that is said*] If not mentioned; **Probe**: What have you heard about, Secret rooms, Toilet/Latrines, freemason etc? How does (*each mentioned*) make you feel?<br>• Did you hear any stories about this house or the other project houses? What was said? [***Interviewer:*** *List all that is mentioned and probe for each especially if mentioned for the first time. Take note of: Secret room, sacrifices, children death, health insurance cover, freemason*] When did you hear about that (before or after moving)?<br>• Who told you those stories?<br>• Why do you think they told those stories?<br>• Does living in this house make you or your family members like outcasts or discriminated in your community/village? Why yes or not? Why do you think they have been saying what they say? What have you been telling them in return? What have they been telling you in return (if different from what has been mentioned earlier)?<br>• Do your neighbors or other community members who are **NOT** living in similar houses visit you? If no, is it different now

	compared to when you were living in your previous house? What do you think is the reason for the difference [*if not yet mentioned up to now*]?<br>• How do you handle your neighbors' or other community members' opinions about the house? **Probe:** How do you cope with that? How do you help your family to cope with that?<br><br>**[A short script ONLY if people do not open up about any of the prevailing myths]**<br><br>"*We have heard from people in other villages in this area that, other villagers who did not get a chance to receive the star houses are envy over those who received the houses*"<br><br>• What can you say about this?<br>• What do you think about it?
**PERSONAL REFLECTIONS ABOUT THE HOUSES**	• Based on your experience living in the house, what do you see as advantages to living in the house? Please, tell us about the advantages.<br>• Based on your experience living in the house, what do you see as disadvantages to living in the house? Please, tell us about the disadvantages.<br>• Do you see more or fewer insects/mosquitoes/flies in the house? In which areas?<br>• From your experience living in the house, what information can you suggest to be told to your neighbors or other community members about these houses? [***Interviewer:*** *Encourage the respondent to keep on mentioning*] **Probe**: Who do you think should tell them about such info? What do you think is the best way to reach your neighbors or other community members with such information? Which ways of delivering such information do you think people in this community do not prefer? Why do they not prefer [***Interviewer:*** *Probe for each way mentioned, if any*]
**Outside space**	• Where do you greet visitors to your home?<br>• Do you often sit outside the house?<br>• Do you sit outside in the evening or at night?<br>• When do the children go indoors at night?<br>• Would you change anything about the design of the outside sitting area?<br>• Do you keep doors open in the evening or always keep them closed?

**Downstairs**	• What activities do you do in the downstairs area?<br>• When do you use the downstairs area?<br>• How often do you cook inside?<br>• What do you think about the stove?<br>• How often do you eat inside?<br>• Do you use the storage area to store food? What else do you store in this space?
**Upstairs sleeping areas**	• Who sleeps upstairs?<br>• Do they children always sleep upstairs in the bedroom? If not, where else do they sleep?<br>• What time do the children go to bed?<br>• Do the children sleep under bednets?<br>• Do the nets have holes in them?<br>• When did you last replace the nets?<br>• How is the temperature at night?
**Design elements**	• What do you think about the netting?<br>• …the stove?<br>• …the floor?<br>• …the storage area?<br>• …water system?<br>• …latrine?<br>• …doors?<br><br>Would you change anything about these parts of the house if you could? If so, what would you change?
**Maintenance and cleaning**	• Have you had to fix anything in the house in the last six months?<br>• What? Who did the work? How long did it take? How much did it cost?<br>• Did you have to do any maintenance in the rainy season? What? Who did the work? How long did it take? How much did it cost?<br>• What about the dry season? What? Who did the work? How long did it take? How much did it cost?<br>• Do you share any maintenance work with relative, neighbours or friends?<br>• Do you see a difference with star home compared to previous home? Is more or less maintenance needed? What makes you

	say that?<br>• How often do you sweep the floor? Downstairs? Upstairs?<br>• Do you clean more or less compared to the previous house? Why?
**CLOSING**	• What else that we have not talked about star homes project that you want to share with me?<br>• Do you have any questions?<br><br>**Interviewer**: Give an overall summary of the discussion and thank the respondent for his/her time

END TIME: |___|___| : |___|___| am/pm
