# Supplementary material for: Community responses to a novel house design: A qualitative study of “Star Homes” in Mtwara, southeastern Tanzania
Source: PLoS One. 2025 Jan 22;20(1):e0309518. doi: 10.1371/journal.pone.0309518 (PMC11753654; doi:10.1371/journal.pone.0309518)
Supplement: S3 Table — (PDF) [file pone.0309518.s003.pdf]

Supporting Information Table 3: Codes, themes and quotes.

Codes	Themes	Sample quotes
Challenges to participation; Free Health Care; Free Mattress; Frequent visits; Gifts and expectations; Star House; Comfort	Overall comfort of Star Homes	*Other significance is [that], for us [we are] living [a] comfortable life than those who live into comparison houses.*<br><br>*I can't hide that [the fact that these Star Homes are comfortable]; the children are sleeping into nice places even supportive [and even have support] for their back.*<br><br>*It is an assurance of sleeping with my children into a comfortable place, I receive all the services. When a child [get] got sick, we take him/her and receive some prescription and [get] got medicines.*
Renovation and maintenance; Door; Lights; problems; Shade nets; Roof	Entering and leaving the dwelling	*They [children] can enter in the house without closing the door even though I told them to close.*<br><br>*Some other time when the 3 years old baby wakes up, he/she [leave] left the door exposed and I close it when I reach at home.*<br><br>*The lock of the door doesn't support an opening [the lock has been broken and requires repair].*
Free mosquito nets; Mosquito net use; Bed nets; protection from malaria;	Sleeping inside	*The shed nets are good in short [brief], they are nice and designed well.*<br><br>*The only challenge here is only water penetrating [ingressing from the shade nets] inside the house.*<br><br>*Because of going outside now and then [Because we have to go in and out with children], to take care of young child is challenging sometimes may*

		poo, you need water to clean so she will need to go down to throw faeces in latrine, that is why we decided to sleep downstairs
Cooking; Stove; Toilet; Ventilation; Water;	Water, sanitation and cooking	*We have plenty of water for free….. that tank [water tank] is durable*<br><br>*We have a permanent toilet. So, they [neighbors] see that although they have been given something.*<br><br>*Yes, we cook there [at traditional house] because cooking cassava ugali is difficult [in this stove].*
Jealousy; Beliefs; support from neighbours	Response of the wider community	*Yes, the neighbors, even out of my visibility some of them they say, we're being observed like local chicken and large group declares that, we are very lucky to be in the project.*<br><br>*[Referring to the statement above on why they articulated as such] It is because they are jealous of us.*<br><br>*[On why they talk like that?] There is a difference between us and them.*
**Note:** The codes and themes were interlinked and burrowed the data and quotes from each other.		
